# Constructing models for Crohn's disease diagnosis and prediction of infliximab non-response based on angiogenesis-related genes

**DOI:** 10.3389/fimmu.2024.1239496

**Published:** 2024-01-26

**Authors:** Chenwei Zheng, Xiangbo Chen, Yujing Ke, Xiaolin Xu, Chao Wu, Lingling Jiang

**Affiliations:** ^1^ School of Clinical Medicine, Fujian Medical University, Fuzhou, Fujian, China; ^2^ Department of Gastroscopy, Fujian Medical University Affiliated First Quanzhou Hospital, Quanzhou, Fujian, China

**Keywords:** Crohn’s disease, bioinformatics analysis, machine learning, infliximab, angiogenesis, prediction model

## Abstract

**Background:**

Angiogenesis response plays a crucial role in the occurrence and development of Crohn’s disease (CD) and may involve the mechanism of infliximab non-response. However, the role of angiogenesis-related genes in Crohn’s disease has not been comprehensively studied. This study aimed to explore the expression profiles of angiogenesis-related genes in CD patients and construct models for disease diagnosis and prediction of infliximab non-response.

**Methods:**

CD-related microarray datasets were collected from the GEO database. Unsupervised consensus clustering analysis was performed based on differentially expressed angiogenesis-related genes to divide CD samples into two distinct clusters. Weighted gene co-expression network analysis (WGCNA) was conducted on the clusters to identify angiogenesis-related module. Based on the differentially expressed genes in the module, machine learning algorithms were employed to further identify hub genes and construct a disease diagnostic model. Subsequently, treatment outcome-related genes were extracted from these hub genes, and a predictive model for infliximab non-response in CD patients was ultimately built.

**Results:**

Based on angiogenesis-related genes, we identified two distinct CD clusters (C1 and C2). Compared to C1, the metabolic pathways in C2 were significantly upregulated, and there was a higher abundance of cell clusters such as M1 macrophages and plasma cells. Additionally, C2 showed a poorer response to infliximab. Furthermore, a predictive model for infliximab non-response in CD patients was constructed based on the hub genes, and it was successfully validated using an external dataset.

**Conclusion:**

Comprehensive analysis of angiogenesis-related genes revealed different clusters of CD, which exhibited differential response rates to infliximab. The construction of models provides a reference for disease diagnosis and drug selection, aiding in clinical decision-making.

## 1 Introduction

Crohn’s disease (CD) is a subtype of inflammatory bowel disease (IBD) that has a chronic and relapsing-remitting course, which is influenced by genetic, environmental, and microbial factors (1, 2). The incidence of CD is increasing in emerging industrialized countries in Africa, Asia, and South America (3). Although anti-TNF drugs like infliximab are effective in treating the majority of Crohn’s disease patients (2, 4), up to 30% of patients experience primary non-response to infliximab therapy (5). This increases the risk of adverse drug reactions and imposes a significant economic burden on the patients. However, the reasons behind treatment failure have yet to be fully understood.

Angiogenesis is a characteristic feature of chronic inflammation (6), and microvascular angiogenesis is profoundly influenced by inflammation in IBD (7, 8). This process is closely associated with the pathogenesis of IBD and disease severity (9–11). Furthermore, vascular endothelial growth factor (VEGF), a protein related to angiogenesis, has been reported as a predictive marker for the response to infliximab therapy. Patients with higher baseline levels of VEGF in their blood exhibit a poorer response to infliximab treatment (12). Despite this, there is still a limited understanding of angiogenesis-related genes in the intestinal mucosa of CD patients.

In this study, we aimed to comprehensively investigate the relationship between angiogenesis-related genes and Crohn’s disease (CD). Based on differentially expressed angiogenesis-related genes, we divided CD samples into two clusters (C1 and C2) and analyzed their distinct biological characteristics and response to infliximab treatment. Using machine learning methods, we identified hub genes involved in disease occurrence, development, and angiogenesis. We then constructed predictive scoring systems for disease diagnosis and infliximab treatment response based on these hub genes. We hope that our research will contribute to the advancement of personalized treatment strategies for Crohn’s disease.

## 2 Methods

### 2.1 Data collection

**Figure 1:**
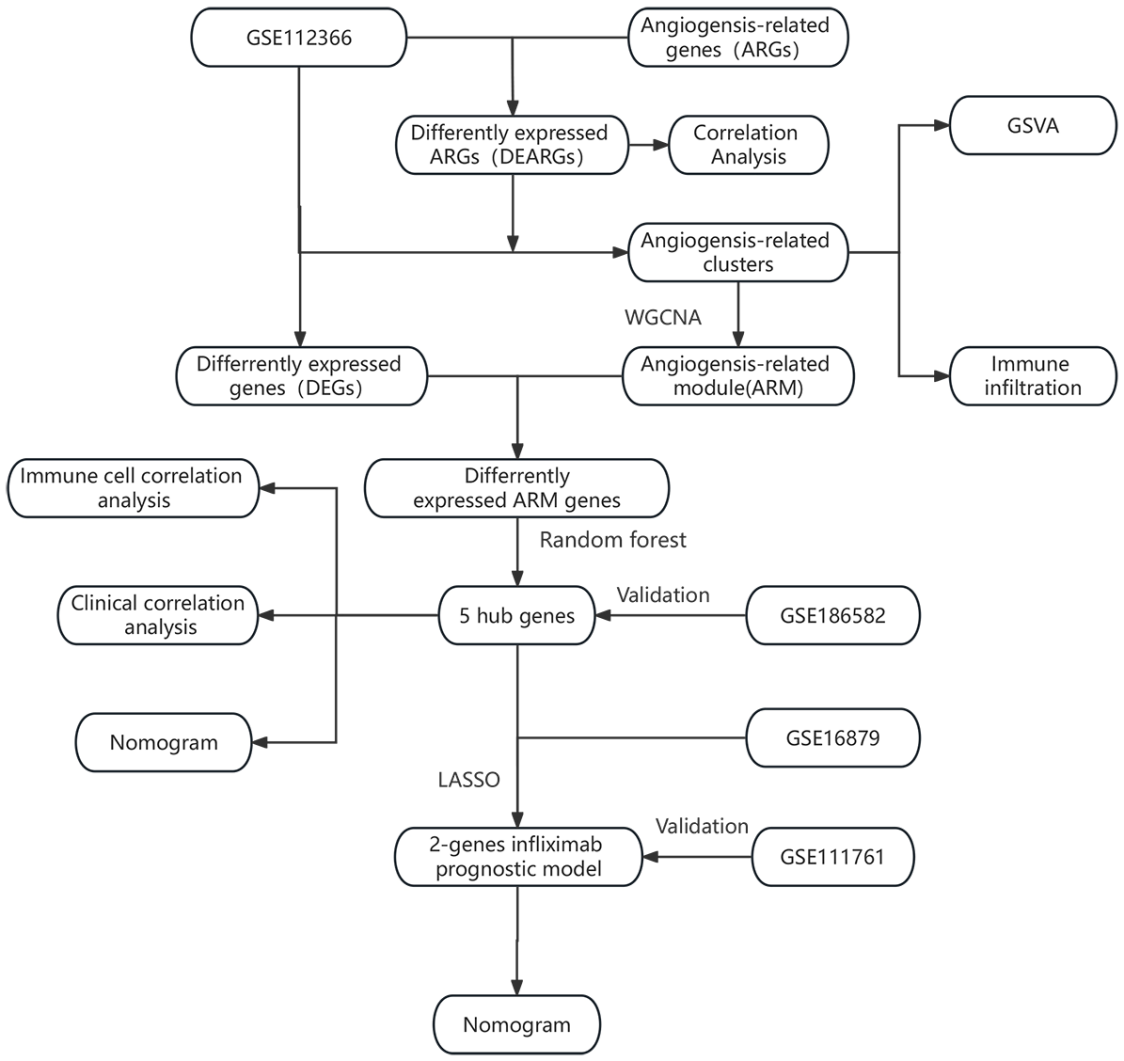
Overall flowchart for the study.

**Figure 1** illustrates the workflow of our study, which involved collecting relevant datasets of intestinal mucosal biopsies from Crohn’s disease (CD) patients from the GEO database (13). The datasets included GSE112366 (362 CD samples; 26 control samples) (14), GSE186582 (138 CD patients without anti-TNF treatment; 25 controls) (15), GSE16879 (20 CD patients with response to infliximab; 17 CD patients with no response to infliximab) (16), and GSE111761 (3 CD patients with response to infliximab; 3 CD patients with no response to infliximab) (17). The datasets were normalized and log2-transformed using the R package “limma” (18). The hallmark gene sets are provided by the Molecular Signatures Database (MSigDB). They consist of carefully curated 50 gene sets, representing a wide range of biological processes or pathways. So we downloaded the angiogenesis-related hallmark gene set (HALLMARK_ANGIOGENESIS) from the MSigDB, which includes 36 genes (19).

### 2.2 Identification of differentially expressed genes related to angiogenesis and gene expression correlation analysis

The R package “limma” was used to screen a set of angiogenesis-related genes (HALLMARK_ANGIOGENESIS) consisting of 36 genes. We set a significance threshold with a p-value < 0.05 to identify differentially expressed angiogenesis-related genes between 362 CD samples and 26 control samples in the GSE112366 dataset (18). Subsequently, we calculated the Pearson correlation coefficients among the identified genes using the “cor” function and visualized the results using the R package “corplot.”

### 2.3 Unsupervised consensus clustering analysis

Unsupervised consensus clustering analysis was performed based on the 15 differentially expressed angiogenesis-related genes. The reason we performed this process was to distinguish CD angiogenesis-related clusters. The R package “ConsensusClusterPlus” was used to assign consensus scores to 362 CD samples in GSE112366 and perform clustering based on the optimal number of clusters (20). The quality of clustering was confirmed using principal component analysis (PCA), and the expression level of the screened angiogenesis-related genes in different clusters were visualized using the R package “pheatmap”.

### 2.4 Enrichment analysis and immune infiltration analysis

To better understand the molecular biological characteristics of two different CD clusters, we conducted enrichment analysis on clusters based on the hallmark gene sets (h.all.v2023.1.Hs.symbols.gmt) downloaded from the MSigDB. Single-sample gene set enrichment analysis was conducted using the R package “GSVA” to obtain enrichment scores (21). Enriched terms with a p-value < 0.05 were considered statistically significant. The enriched pathways were visualized using bar plots. The CIBERSORT algorithm was used to analyze the immune cell infiltration in different clusters (22).

### 2.5 Identification of Crohn’s disease hub genes and construction of diagnostic model

To further explore valuable genes associated with angiogenic responses in CD, we utilized the R package “WGCNA” to perform weighted gene co-expression network analysis (23). Based on this analysis, modules related to CD clusters were identified. Since the two CD clusters were identified based on angiogenesis-related genes, the module with the highest correlation and smallest p-value was defined as the angiogenesis-related module (ARM). The differentially expressed genes between CD patients and healthy controls were identified using the online analysis tool GEO2R from the GEO database (13). The differentially expressed genes and the genes in the angiogenesis-related module were intersected to obtain the differentially expressed angiogenesis-related module genes. These genes are not only associated with the pathogenesis of CD but also linked to angiogenesis, hence holding value for further research.

Based on the identified genes, we compared the areas under the receiver operating characteristic (ROC) curve (AUC) of three machine learning algorithms: generalized linear model (GLM), random forest (RF), and support vector machine (SVM), to identify the algorithm with better classification performance (24, 25). The ROC curve is a graphical representation used to illustrate the trade-off between sensitivity and specificity of a binary classification model at different thresholds, while AUC is a numerical measure that quantifies the entire ROC curve. It is a commonly used metric for evaluating the performance of binary classification models. The training set comprised 70% of the sample size, while the validation set comprised 30%. Upon comparison, the RF algorithm exhibited superior performance. Therefore, in this study, we utilized the R software package ‘randomForest’ to identify the top 5 most meaningful genes as hub genes related to the occurrence and development of CD and associated with angiogenesis. Based on these 5 genes, we split the dataset into a training set (70%) and a validation set (30%). Subsequently, a disease diagnostic model was constructed. Finally, we evaluated the model’s performance in the GSE186582 dataset, and the confidence interval (CI) of AUC was calculated using the Bootstrap method.

### 2.6 Immune cell correlation analysis and clinical correlation analysis

For gaining a deeper insight into the influence of hub genes, spearman correlation analysis was performed to assess the correlation between the identified hub genes and the infiltration of immune cells in CD patients, as well as the Crohn’s disease simplified endoscopic score (SES-CD). The SES-CD is a scoring system developed for assessing the severity of CD under endoscopy, which has good clinical applicability (26). A p-value < 0.05 was considered statistically significant. The results were visualized using the R packages “ggplot2”, “ggpubr”, and “ggExtra”.

### 2.7 Constructing a predictive model for infliximab non-response

In order to investigate the response to infliximab therapy in different CD clusters, the CD patients from the dataset GSE16879 were also divided into two clusters (C1 and C2) based on the differentially expressed angiogenesis-related genes. The difference in the response to infliximab between the two clusters were compared using Fisher’s exact test. If the p-value was < 0.05, it was considered statistically significant. Based on the previously identified hub genes, Lasso regression analysis was performed to further identify the characteristic genes associated with different responses to infliximab and construct a predictive model for infliximab non-response. This process was implemented using the R package “glmnet” (27). The nomogram was constructed using the R package “rms.” The predictive performance of the model was demonstrated using AUC, and the stability of the model was further validated in the dataset GSE111761.

## 3 Results

### 3.1 Identification of differentially expressed genes related to angiogenesis and gene expression correlation analysis

**Figure 2:**
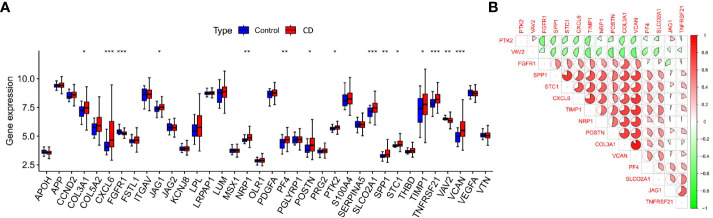
Expression and co-expression analysis of angiogenesis-related genes. **(A)** Differential expression of angiogenesis-related genes in CD samples and control samples, *p < 0.05, **p < 0.01 and ***p < 0.001). **(B)** Co-expression analysis of differentially expressed angiogenesis-related genes.

We compared the differential expression of 36 angiogenesis-related genes between the CD intestinal mucosal tissues and normal controls. Among them, the expression differences of 15 genes (*COL3A1*, *CXCL6*, *FGFR1*, *JAG1*, *NRP1*, *PF4*, *POSTN*, *PTK2*, *SLCO2A1*, *SPP1*, *STC1*, *TIMP1*, *TNFRSF21*, *VAV2*, *VCAN*) were statistically significant (**Figure 2A**). We defined them as differentially expressed angiogenesis-related genes (DEARGs) and used them for subsequent analysis. Co-expression analysis of genes revealed regulatory relationships among DEARGs, with most of the genes showing positive regulation (**Figure 2B**).

### 3.2 Clustering of Crohn’s disease patients based on DEARGs

**Figure 3:**
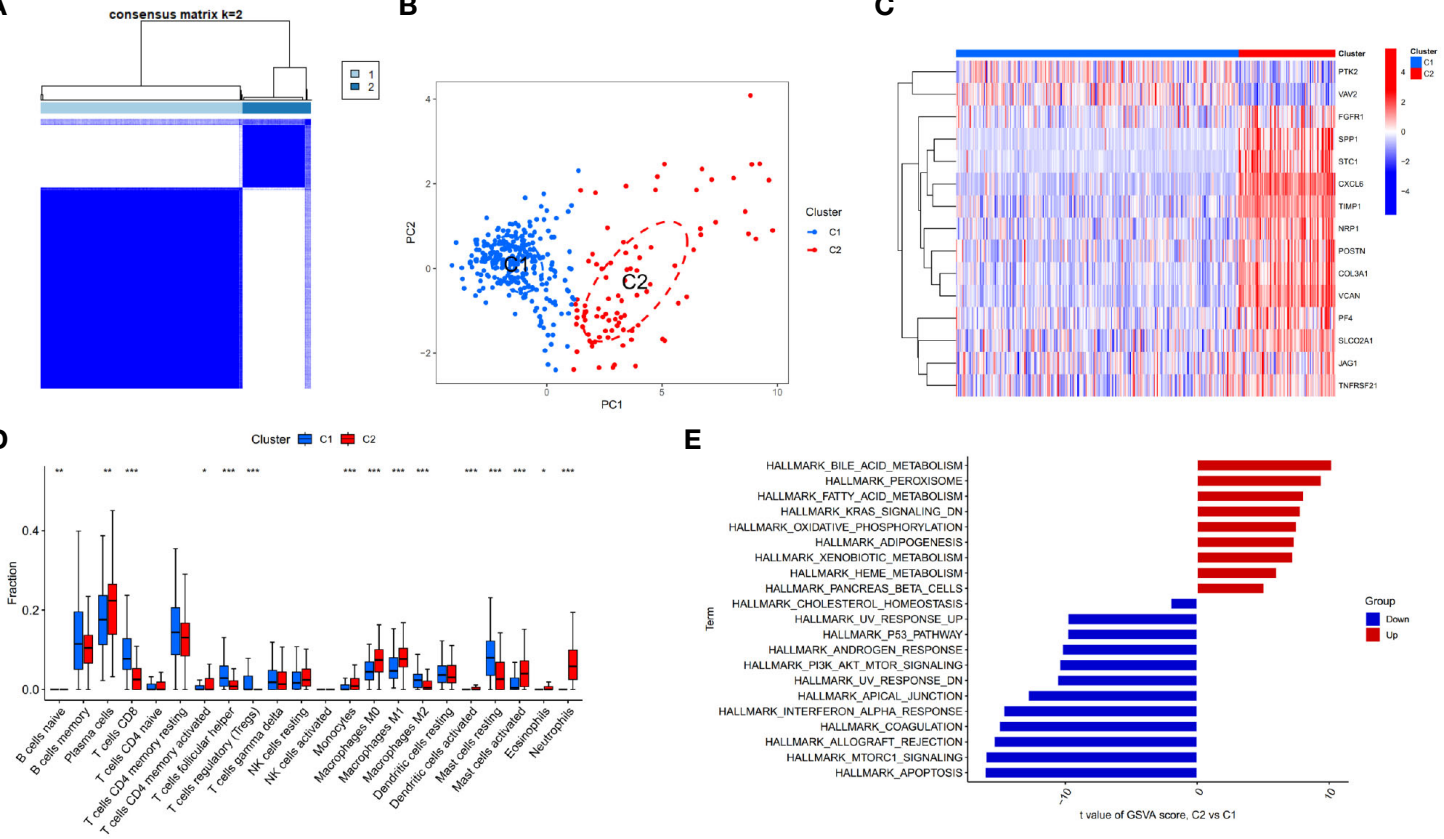
Identifying different clusters of Crohn’s disease. **(A)** Consensus matrix of Crohn’s disease samples in GSE112366. **(B)** PCA showing the degree of distinction between different Crohn’s disease clusters (C1 and C2). **(C)** Heatmap displaying the expression of differentially expressed angiogenesis-related genes in Crohn’s disease clusters (C1 and C2). **(D)** Immune cell infiltration abundance in C1 and C2 clusters (*p < 0.05, **p < 0.01 and ***p < 0.001). **(E)** GSVA enrichment analysis of C1 and C2 clusters.

Based on the identified DEARGs, we divided CD samples into two clusters. As shown in **Figures 3A**, **B**, this clustering method effectively distinguished different clusters of Crohn’s disease (C1 and C2). Subsequently, we compared the expression of DEARGs between the two clusters and found that *PTK2* and *VAV2* were downregulated in the C2 cluster, while the rest of the genes involved in positive regulation of angiogenesis were upregulated (**Figure 3C**).

### 3.3 GSVA enrichment analysis and immune infiltration analysis of CD clusters

GSVA enrichment analysis of different CD clusters revealed that the metabolism and oxidative reactions were more active in C2 cluster, with the upregulation of bile acid metabolism being the most significant. Meanwhile, C1 cluster was associated with pathways involving cell proliferation, apoptosis, and type I interferon response (**Figure 3E**). The study also analyzed immunocyte infiltration and discovered differences in the expression of most immune cells between the two clusters. Neutrophils, activating mast cells, M1 macrophages, plasma cells, and monocytes were more abundant in C2 cluster, while CD8+ T cells, regulatory T cells, M2 macrophages, and resting mast cells showed higher abundance in C1 cluster (**Figure 3D**).

### 3.4 Identification of hub genes and construction of diagnostic model

**Figure 4:**
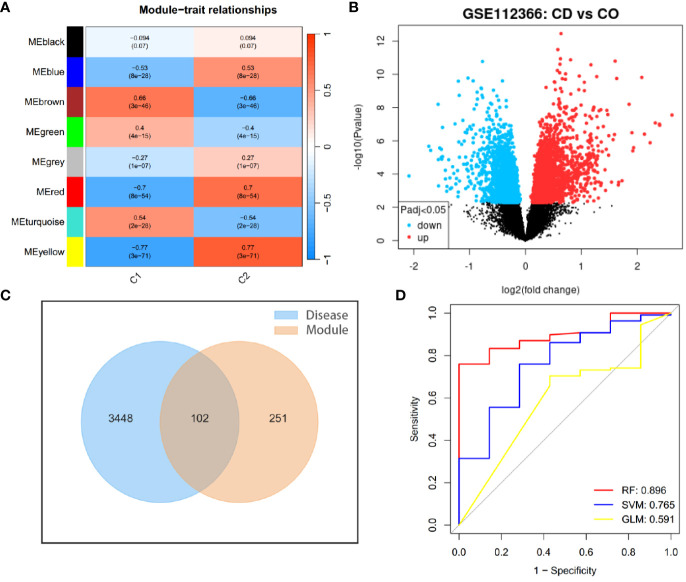
Identifying genes involved in the development of Crohn’s disease and angiogenesis. **(A)** WGCNA module trait for C1 and C2 clusters. **(B)** Volcano plot showing gene expression differences between Crohn’s disease samples and controls. **(C)** Intersection of differentially expressed genes between CD samples (Disease) and controls with characteristic module genes of CD clusters (Module). **(D)** ROC curves illustrating the diagnostic performance of three machine learning algorithms for the disease.

**Figure 5:**
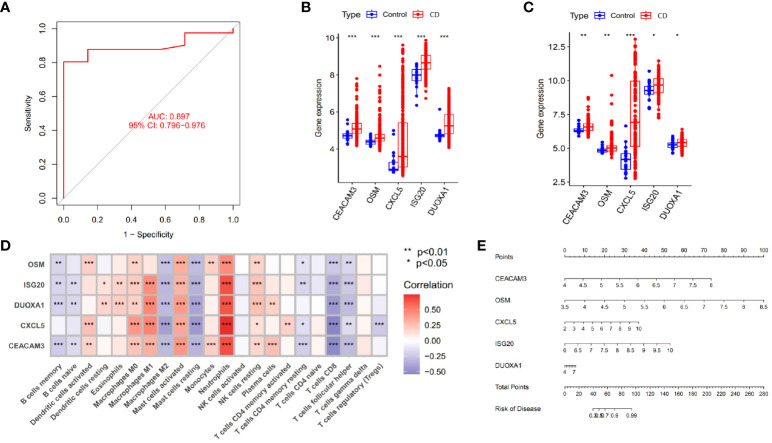
Identification of hub genes and construction of diagnostic models. **(A)** Validation of the diagnostic performance of hub genes in GSE186582. **(B, C)** Differential expression of hub genes between Crohn’s disease patients and controls in GSE112366 **(B)** and GSE186582 **(C)** (*p < 0.05, **p < 0.01 and ***p < 0.001). **(D)** Correlation between hub genes and immune cell infiltration abundance (***p < 0.001). **(E)** Nomogram for the diagnosis of Crohn’s disease.

In the study of CD samples, a Weighted Gene Co-expression Network Analysis (WGCNA) was conducted which revealed that the yellow module “MEyellow” (**Figure 4A**) had the highest correlation with the C2 cluster (R=0.77) and the smallest p-value (3e-71). This indicated that the yellow module was the characteristic module of the C2 cluster. In other words, the genes included in this module were the most distinguishing factors between the C1 and C2 clusters. Since C1 and C2 clusters were differentiated based on differences in angiogenesis-related genes, this suggested a correlation between the genes in the yellow module and angiogenesis response. Therefore, we defined this yellow module as the angiogenesis-related module (ARM). By intersecting the genes in the module with the differentially expressed genes between CD samples and control samples (**Figure 4B**), we obtained 102 differentially expressed angiogenesis-related module genes (**Figure 4C**). These genes were not only involved in the development of the disease but also correlated with angiogenesis. Based on these genes, we compared the diagnostic performance of three machine learning algorithms: random forest (RF), support vector machine (SVM), and generalized linear model (GLM). Our findings suggested that the RF algorithm was more effective in identifying CD patients (**Figure 4D**). Additionally, we identified the top 5 significant genes (*CEACAM3*, *OSM*, *CXCL5*, *ISG20*, *DUOXA1*) as hub genes using the RF algorithm. These genes were found to be upregulated in CD patients in both GSE112366 and GSE186582 datasets (**Figures 5B, C**). We constructed a diagnostic nomogram (**Figure 5E**) based on these hub genes, and we demonstrated the excellent performance of the model in the external dataset GSE186582 (AUC=0.897, 95%CI:0.796-0.976) (**Figure 5A**).

### 3.5 Analysis of immune cell correlation and clinical relevance

**Figure 6:**
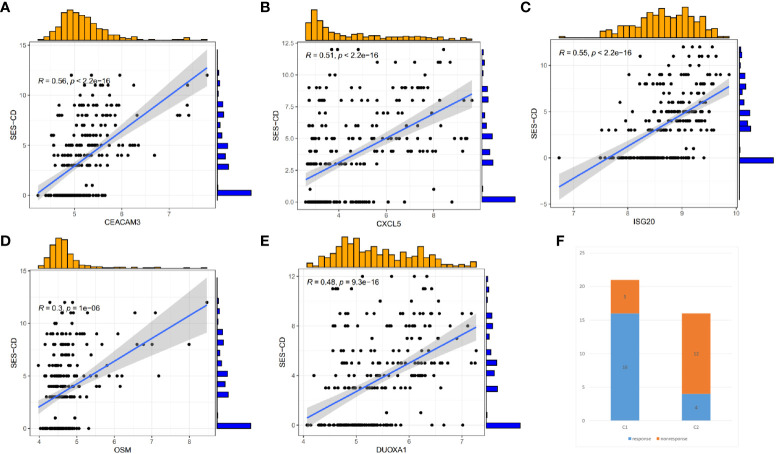
Correlation between hub Genes and SES-CD, and efficacy differences of infliximab in Crohn’s disease patients. **(A–E)** Correlation of 5 hub genes with clinical SES-CD. **(F)** Response of Crohn’s disease patients to infliximab.

As shown in **Figure 5D**, the 5 identified hub genes were positively correlated with neutrophil and activating mast cell abundance and negatively correlated with CD8+ T cell, M2 macrophage, and resting mast cell abundance. Among them, *CXCL5* showed the strongest positive correlation with neutrophils and the strongest negative correlation with CD8+ T cells. Clinical relevance analysis indicated that the expression levels of these 5 hub genes were positively correlated with endoscopic SES-CD scores. This suggested that higher expression levels of hub genes were associated with increased disease severity (**Figures 6A–E**).

### 3.6 Construction of infliximab non-response prediction model

**Figure 7:**
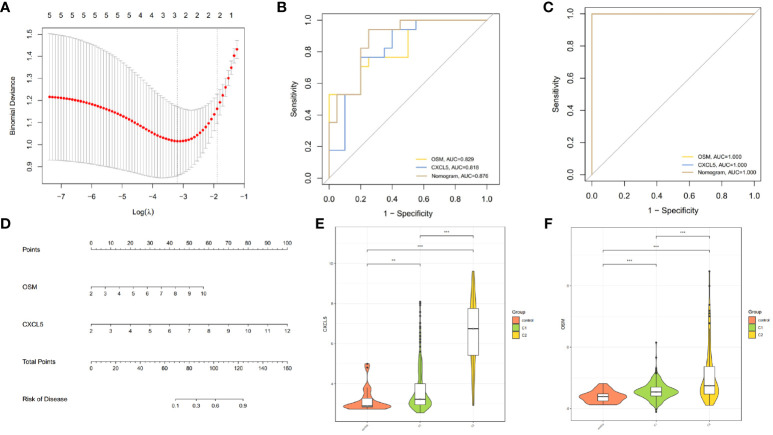
Construction of predictive model for infliximab non-response. **(A)** Cross-validation for tuning parameter selection using LASSO regression. **(B, C)** ROC curves demonstrating the ability of *OSM*, *CXCL5*, and the nomogram to predict infliximab non-response. **(D)** Nomogram for predicting infliximab non-response in CD patients. **(E, F)** Differential expression of *CXCL5* and *OSM* among controls, C1 cluster, and C2 cluster in GSE112366 (**p < 0.01 and ***p < 0.001).

To understand whether there are differences in the response to infliximab among different CD clusters, we also divided the patients in the GSE16879 dataset into C1 and C2 clusters. We then compared the response to infliximab between different clusters and found that in the C1 cluster, 16 patients responded to infliximab, while 5 patients did not respond. In the C2 cluster, 4 patients responded, and 12 patients did not respond (**Figure 6F**). Using Fisher’s exact test, we obtained a p-value <0.05, indicating that the difference in response to infliximab between the two clusters was statistically significant. Our findings suggested that there was a higher probability of infliximab non-response in the C2 cluster. As a subset of cluster characteristic module genes, hub genes may be associated with infliximab non-response. Therefore, based on the identified hub genes, we further used Lasso regression analysis to identify the feature genes that could predict infliximab response (**Figure 7A**). Among the 5 hub genes, *CXCL5* and *OSM* were identified as being associated with infliximab non-response, and both had good predictive performance (*CXCL5*, AUC=0.818; *OSM*, AUC=0.829) (**Figure 7B**). We compared the differences in *OSM* and *CXCL5* between non-responders and responders before and after treatment. We found that *OSM* did not exhibit statistically significant differences before and after treatment. In contrast, *CXCL5* showed a certain degree of decrease after treatment. However, even after treatment, the levels of *CXCL5* in non-responders remained higher than those in responders (**Supplementary Material**). Based on *CXCL5* and *OSM*, we constructed a prediction model for infliximab non-response (AUC=0.876) and presented it as a nomogram (**Figure 7D**). The model was validated in the external dataset GSE111761 (**Figure 7C**). Expression differences of *CXCL5* and *OSM* genes in the controls, C1 cluster, and C2 cluster of the gene set GSE112366 were visually demonstrated in **Figures 7E, F**.

## 4 Discussion

Crohn’s disease is a chronic heterogeneous disease with diverse manifestations and progressions, making its diagnosis inherently challenging (28). Moreover, patients often exhibit varied responses to therapeutic drugs, with around 30% of patients showing primary non-response to infliximab treatment (5). This subgroup of patients faces increased economic burden and risks of medication side effects. Therefore, selecting the most effective medications is of paramount importance for these patients.

In this study, we focused on angiogenesis as a starting point to explore the heterogeneity of CD patients and developed models for CD diagnosis and infliximab non-response based on hub genes. The findings of this study may contribute to a better understanding of Crohn’s disease among researchers and provide valuable insights for clinical treatment.

Based on the expression of angiogenesis-related genes, we successfully identified different clusters of Crohn’s disease (C1 and C2). Compared to the C1 cluster, the C2 cluster showed upregulation of multiple metabolic pathways, and poorer response to infliximab. Similar to our findings, Matthew Weiser et al. also identified two subtypes of Crohn’s disease (colonic and ileal), with the ileal cluster showing significantly upregulated metabolic pathways, including lipid metabolism (29). Studies have shown that endothelial cells (ECs) can metabolize fatty acids to acetyl-CoA to maintain the tricarboxylic acid (TCA) cycle and promote deoxynucleotide triphosphate (dNTP) synthesis, together with non-nucleotide substrates, to facilitate EC proliferation (30). This suggests that the Crohn’s disease cluster with active lipid metabolism may exhibit a higher degree of angiogenesis.

Further investigation revealed that the C2 cluster with active angiogenesis showed a higher rate of non-response to infliximab. This suggests that angiogenic responses may play a role in the mechanisms of infliximab non-response. A study by Alicia Algaba et al. found that elevated baseline levels of vascular endothelial growth factor (VEGF) are associated with poor response to anti-TNF treatment and VEGF can serve as a useful biomarker for anti-TNF treatment response (AUC=0.8) (12). This also suggests a potential connection between angiogenesis and anti-TNF treatment. In our exploration of the expression of angiogenesis-related genes in Crohn’s disease patients, we found upregulation of *OSM*, *CXCL5*, *CEACAM3*, *ISG20*, and *DUOXA1* genes in Crohn’s disease patients. Among them, two genes, *OSM* and *CXCL5*, serve as good predictive markers for infliximab non-response (*OSM*, AUC=0.829; *CXCL5*, AUC=0.818). The finding was also validated in an external dataset, further supporting the relationship between angiogenesis and anti-TNF treatment response.

According to studies, Oncostatin M (OSM) belongs to the interleukin-6 (IL-6) family and has the same receptor subunit, gp130, as IL-6 and leukemia inhibitory factor (LIF) (31, 32). OSM has been found to induce angiogenesis both *in vivo* and *in vitro*, and its effect on angiogenesis is stronger than that of IL-6 and LIF. OSM can stimulate vascular smooth muscle cells to produce VEGF through various pathways such as PI3K, p38 MAPK, Erk1/2, STAT1, and STAT3, whereas other gp130 ligands have not been reported to regulate VEGF mRNA. However, it is important to note that promoting VEGF secretion is not the sole mechanism by which OSM promotes angiogenesis, as VEGF-neutralizing antibodies only inhibit 21% of *OSM* angiogenesis effects (33, 34).

The expression of the *OSM* gene is closely related to IBD. Nathaniel R. West et al. discovered that *OSM* is overexpressed in the tissues of active IBD patients and is strongly associated with the severity of tissue pathology (35), which is consistent with our findings. Furthermore, some studies also found a close correlation between baseline *OSM* expression and poor response to infliximab (36–38). Compared to responders to anti-TNF therapy, *OSM* is one of the top 20 genes most strongly expressed in non-responders to anti-TNF therapy. In addition, neutralizing *OSM* was found to significantly alleviate colitis in mice. Therefore, *OSM* has the potential to serve as both a biomarker and therapeutic target for IBD (35), which further bolsters our own research findings.

Similar to OSM, CXC motif chemokine ligand 5 (CXCL5) also plays a role in promoting angiogenesis (39). CXCL5, also known as epithelial neutrophil-activating peptide 78 (ENA-78), is a member of the ELR (+) CXC chemokine family and can mediate angiogenesis in the absence of inflammation. CXCR2 is the receptor for CXCL5 and mediates the angiogenic effects of ELR (+) CXC chemokines (40). Research has demonstrated that CXCL5 is involved in the angiogenic effects of multiple diseases. It orchestrates the modulation of FOXD1 expression through the activation of the AKT/NF-κB pathway, thereby fostering angiogenesis in colorectal cancer (41). It is also a crucial angiogenic factor in idiopathic pulmonary fibrosis (42). In gastric cancer, it can significantly impact the vascular distribution of tumors (43). In renal cell carcinoma, androgen receptor mediated CXCL5 signaling activates the AKT/NF-κB pathway to facilitate angiogenesis (43, 44). Blocking CXCL5 can effectively reduce tumors blood supply (45).

K Z’Graggen et al. found that CXCL5/ENA-78 is preferentially expressed in intestinal epithelial cells in IBD. Although CXCL5/ENA-78 protein is also present in some normal controls, CXCL5/ENA-78 mRNA is completely negative (46). In addition, the study also pointed out that CXCL5 may be involved in the early stages of IBD and may contribute to the continuous recruitment and activation of neutrophils (46). John H Kwon’s experiments showed that pretreatment with antisense oligonucleotides against lipopolysaccharide-induced CXC chemokine (LIX) reduced DSS-induced colitis in mice. LIX was identified as a murine homolog of CXCL5/ENA-78. This suggests that *CXCL5* may be a potential therapeutic target for IBD (47). In our study, *CXCL5* can also serve as a good predictive marker for infliximab non-response. This indirectly suggests a potential connection between OSM and CXCL5. Indeed, previous research on pneumonia has shown that OSM plays a significant role in inducing CXCL5 and can directly initiate the mRNA synthesis of CXCL5 (48). However, whether there is a connection between the two in CD patients still requires further experimental validation. In our study, the predictive model constructed by combining *CXCL5* and *OSM* (AUC=0.876) outperforms the predictive capabilities of *CXCL5* and *OSM* individually. Additionally, the predictive performance of this model is also superior to serum VEGF (AUC=0.8).

Furthermore, research has shown that *CEACAM3* is significantly upregulated in pediatric Crohn’s disease patients’ intestinal biopsies. The study also found that various IBD triggers can regulate *CEACAM3*. This suggests that trigger-induced *CEACAM3* may play a role in the pathogenesis of IBD (49). Although there is a lack of research on *DUOXA1* in CD, literature search revealed that one of the splice variants of DUOXA1 can be functionally associated with DUOX2 (50), and in active ulcerative colitis, DUOX2 is significantly upregulated (51). Since Crohn’s disease and ulcerative colitis both belong to inflammatory bowel diseases, *DUOXA1* has potential research value. ISG20 has been reported to be a major effector of innate immune responses against various pathogens, including viruses, bacteria, and parasites, and ISG20 is also involved in angiogenesis in liver cancer (52, 53). However, whether *ISG20* is involved in the pathogenesis of Crohn’s disease requires further research.

The analysis of immune infiltration reveals that the C2 cluster has a higher abundance of M1 macrophages and plasma cells. According to the study by Renaud Gaujoux (54), the abundance of inflammatory macrophages and plasma cells is significantly higher in non-responders to anti-TNFα treatment compared to responders, and this difference persists after treatment. Previous studies have indicated that during intestinal inflammation, an increase in the abundance of inflammatory macrophages can lead to elevated secretion of TNFα at the site of inflammation (55), and elevated levels of TNFα can support the survival of plasma cells (56). Therefore, if both cell subtypes increase simultaneously, it may indicate that pathological tissue has a higher concentration of TNFα. Hence, the elevated levels of TNFα could be one of the reasons for the lower response rate in the C2 cluster.

In terms of clinical applications, a nomogram is a practical and intuitive tool widely employed in predictive models in the field of medicine (57). When prediction is required, one simply needs to locate the value of the corresponding gene on the nomogram, then use connecting lines to find the corresponding scale above, obtaining the individual gene’s points. By summing the individual gene points, the total points are obtained. Locating the corresponding scale for the total points provides the probability of the outcome occurrence. Our study includes two nomograms. For diagnosis, measuring the expression levels of the *OSM*, *CXCL5*, *CEACAM3*, *ISG20*, and *DUOXA1* genes and using the first nomogram allow us to determine the probability of disease occurrence. This contributes to early disease diagnosis and provides opportunities for early intervention, thereby improving treatment outcomes. Meanwhile, the expression levels of *OSM* and *CXCL5* can be further used in the second nomogram to predict the probability of non-response to infliximab. Based on the predictive results, doctors can adjust the treatment plan, such as adjusting dosage, incorporating other therapeutic medications, or changing biologics (58). This aids in avoiding ineffective treatment plans for patients, enhancing the individualized level of treatment, and reducing unnecessary drug exposure and potential side effects. Therefore, our research results are beneficial for both disease diagnosis and decision-making regarding subsequent treatment strategies. Furthermore, predictive models help increase patients’ understanding of their own diseases and treatment plans. Patients can actively participate in medical decision-making and gain a more comprehensive understanding of the risks and benefits of treatment plans.

In summary, our comprehensive analysis of angiogenesis-related genes in CD patients identified two distinct clusters. Further analysis revealed hub genes involved in the pathogenesis of CD, with *OSM* and *CXCL5* being associated with non-response to infliximab. Simultaneously, we constructed disease diagnostic and infliximab non-response predictive models, validating their good performance. This may contribute to the diagnosis and personalized treatment of CD patients.

## 5 Limitations

Although we have examined our research results using multiple datasets and achieved relatively satisfactory validation performance, our study still possesses certain limitations. Firstly, the sample size for investigating the response to infliximab treatment is limited, and the clinical information associated with the samples is inadequate, potentially impacting the precision of our study. Secondly, our study only included microarray data, which may impact the generalizability of the model. Thirdly, our research predominantly relies on database samples and bioinformatics analysis, necessitating further prospective experiments to enhance the robustness of our research findings. Lastly, we observed a correlation between angiogenesis-related genes and the non-response to infliximab in CD patients. However, whether there is a causal relationship between angiogenesis response and the therapeutic effectiveness of infliximab still requires further experimental confirmation.

## Data availability statement

The datasets presented in this study can be found in online repositories. The names of the repository/repositories and accession number(s) can be found in the article/**Supplementary Material**.

## Author contributions

CZ proposed the research topic and designed the study protocol. XC were responsible for the paper writing. YK collected and analyzed the data. XX contributed to the data visualization. CW and LJ reviewed and revised the manuscript. All authors contributed to the article and approved the submitted version.
