# Supplementary figures and images for: Constructing models for Crohn's disease diagnosis and prediction of infliximab non-response based on angiogenesis-related genes
Source: Front Immunol. 2024 Jan 26;15:1239496. doi: 10.3389/fimmu.2024.1239496 (PMC10853379; doi:10.3389/fimmu.2024.1239496)

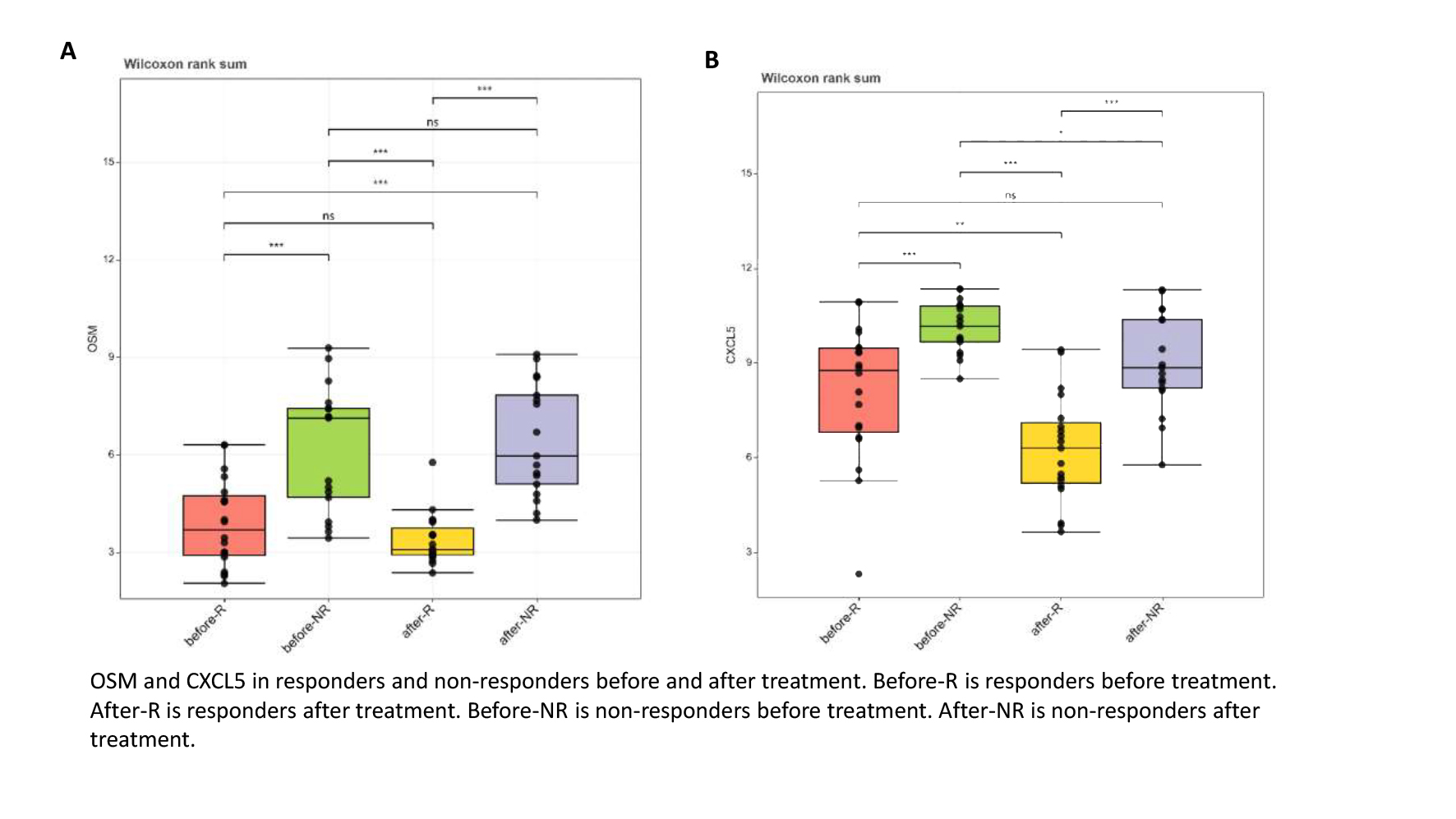

Supplement: Supplementary file 1 [file Image_1.jpeg]
